# Work-Related Musculoskeletal Disorder Prevalence by Body Area Among Nurses in Asia: Systematic Review and Meta-Analysis

**DOI:** 10.3390/ijerph22040652

**Published:** 2025-04-21

**Authors:** Julien Jacquier-Bret, Philippe Gorce

**Affiliations:** 1 University of Toulon, CS60584, CEDEX 9, 83041 Toulon, France; gorce@univ-tln.fr; 2 International Institute of Biomechanics and Occupational Ergonomics, Avenue du Docteur Marcel Armanet, CS 10121, 83418 Hyères Cedex, France

**Keywords:** musculoskeletal disorders, prevalence, body area, nurse, years of experience, nurse-to-bed, continent, meta-analysis, systematic review, healthcare professional

## Abstract

Nurses are highly exposed to work-related musculoskeletal disorders (WMSDs). Several studies on this have been carried out in different Asian countries, but no synthesis was conducted. The aim of this study was to map the evidence of WMSDs among nurses in Asia. A systematic review and a meta-analysis with subgroups were performed during October 2024. Five open databases were scanned without a date limit. The article selection and data extraction processes were performed independently by two reviewers. The results report was conducted according to the Preferred Reporting Items for Systematic Reviews and Meta-Analyses (PRISMA) guidelines. Among the 15,751 unique identified records, 40 studies were included, covering a total of 19,903 Asian nurses. A high heterogeneity (Cochran’s Q test and I^2^ statistic) was evidenced between the studies. The meta-analysis polled an overall prevalence of 84.3% (95% CI: 81.1–87.4%). The lower back (58.4%, 95% CI: 52.9–63.8%), neck (45.7%, 95% CI: 38.1–53.2%) and shoulder (43.0%, 95% CI: 36.2–49.9%) were the three most exposed areas. Subgroup analyses have shown that the presence of WMSD is steadily increasing, and that years of practice reduce the exposure of the neck and shoulder, while the lower back becomes more exposed. Further efforts are needed to prevent WMSDs among Asian nurses, in order to improve their quality of life at work.

## 1. Introduction

Nurses’ professional activities are characterized by heavy workloads, frequent handling of patients and heavy equipment, long working hours and shift rotations, which expose them to work-related musculoskeletal disorders (WMSDs). WMSDs are defined as damage to muscular, tendon, ligament and nerve tissue, leading in the short- to mid-term to pathologies of the musculoskeletal system [1]. These WMSDs are currently the most common occupational injuries among nurses [2]. Lower back [3], neck (cervical axial pain, radiculopathy and myelopathy [4]), shoulder (rotator cuff injuries [5]) and wrist (carpal tunnel syndrome [6]) pain are most often reported by nurses. These disorders often lead to temporary or permanent loss of work capacity and impair their quality of life. Sun et al. [7] found that WMSDs were one of the causes of sick leave leading to a high turnover among employees. In addition, the increasing demand for healthcare services in a rapidly developing economy leads to a relative shortage of nursing staff. As a result, nurses’ workloads have increased, raising the risk of WMSDs. In response, more and more nurses are looking to leave their job [8].

This trend is particularly prevalent in Asia, where there are a large number of active registered nurses, notably in China (5.2 million), India (2.4 million) and Japan (1.5 million). [9]. Numerous studies conducted in different Asian countries have demonstrated a high overall WMSD prevalence, with values over 80% reported in India (81.0% [10]), China (84.0% [11]), Japan (85.5% [12]) and Iran (84.0% [13]). In their meta-analysis, Wang et al. [14] and Saberipour et al. [15] reported an overall prevalence of 79.0% in China and 84.0% in Iran. Studies carried out in different Asian countries have highlighted that the most exposed areas could be the lower back, as reported by Nguyen et al. [16] (44.3%) in Vietnam and Anap et al. [10] (48.2%) in India; the neck, as found by Almahdawi et al. [17] (61.1%) in Jordan and Shafizadeh [18] (62.7%) in Iran; or the shoulder, as evidenced by Lin et al. [19] in China (85.8%) and Smith et al., 2006 [12], (71.9%) in Japan. Two meta-analyses conducted in Iran [20] and China [14] are also relevant. The first ranked the lower back (61.0%), neck (47.8%) and knee (46.5%) as being the three most affected areas from 41 publications, containing a total sample of 16,350 nurses. The second study ranked the neck (58.0%), shoulder (49.0%) and back (35%) as the areas most exposed to WMSDs, based on 23 publications and a sample of 2042 nurses. The reported prevalences indicate that nurses practice high-risk professional activities, due to the repetitive tasks performed over long periods and handling patients or heavy equipment, most often in awkward postures [21].

Although several studies have been published on the overall prevalence and by body area, it is important to synthesize all this information to gain a better understanding of the individual and environmental factors that constitute risk factors in the occurrence of WMSDs in nurses. These data should help supervisors to develop new prevention, education and awareness policies to reduce the risk of WMSDs. For ergonomic researchers, it could provide valuable information for the design and development of new innovative technologies or work environment organizations. In this context, only two studies have been carried out on a continental scale: Kgakge et al. [22] in Africa (scoping review, 29 publications and 6343 nurses) and Gorce et al. [23] in Europe (systematic review and meta-analysis: 12 studies and 5153 nurses). The overall prevalence reported ranged from 57.1% to 95.7%, respectively, and pooled to 87.8% (95% CI: 83.3–92.2%). Kgakge et al. focused their analysis on the prevalence of the lower back, while Gorce et al. pooled the prevalence of nine body areas.

The aim of this study was therefore to assess WMSD levels among nurses in Asia through a systematic review and meta-analysis. As prevalence is directly related to demographic data, a meta-analysis was carried out to provide answers to the following two questions: “Is the prevalence of WMSD significantly influenced by the number of years of nursing experience?” and “Is there an increase in the prevalence of WMSD over the years?”. The results will provide evidence to support practice by improving the occupational environment and quality of work life for Asian nurses.

## 2. Materials and Methods

**Figure 1:**
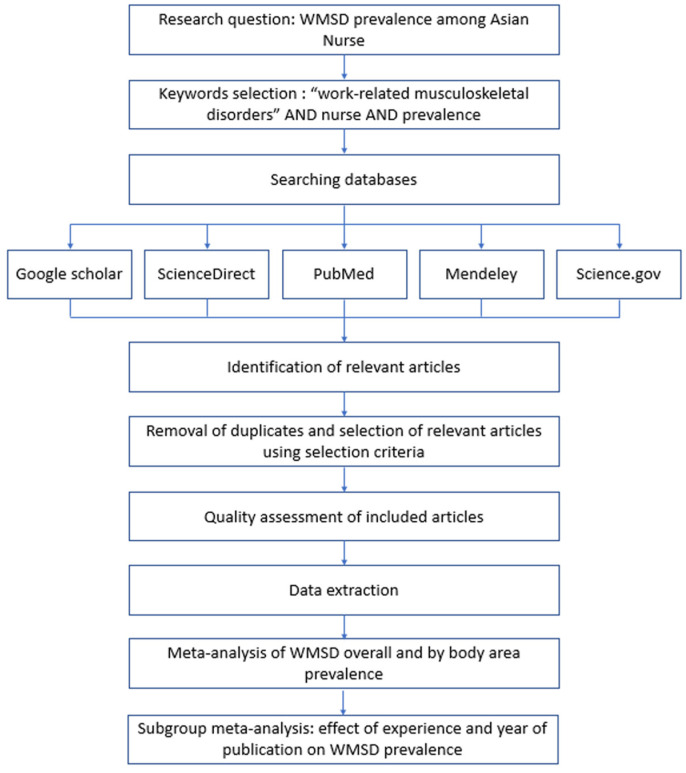
Flowchart of research stages.

The protocol of this systematic review and meta-analysis has been registered at PROSPERO (CRD42024625782). The aim was to perform a comprehensive review of the best available data in order to provide a state-of-the-art synthesis of WMSD prevalence among nurses in Asia. The research was carried out according to the Preferred Reporting Items for Systematic reviews and Meta-Analyses (PRISMA) guidelines [24]. Figure 1 summarizes the different stages of the research process.

### 2.1. Search Strategy

Five free databases were scanned for relevant cross-sectional studies: ScienceDirect, PubMed/Medline, Google Scholar, Science.gov and Mendeley. The research was conducted from 2 October to 31 October 2024. The search was performed using the following combination of keywords in each database: “work-related musculoskeletal disorders” AND nurse AND prevalence. The list of items found in each database was compiled in a single summary file (Excel spreadsheet). Multiple entries were removed using the duplicate search function. Two independent reviewers (P.G. and J.J.-B.) evaluated all studies to select the relevant articles. First, they separately screened titles and abstracts for eligibility based on the inclusion/exclusion criteria. The two selections were compared to identify the final list to be sought for retrieval. A second round was carried out by the two reviewers based on the full text to establish the final list of studies to be included in the analysis. Studies that did not meet the inclusion criteria were excluded. All discrepancies were resolved by consensus and re-review of the articles.

The PECOs (P: Participants; E: Exposures; C: Comparisons; O: Outcomes; s: study design) principle was applied to define the inclusion criteria: (1) Participants: nurses from any specialty or department from Asia. (2) Exposures: WMSD prevalence. (3) Comparisons: not applicable. (4) Outcomes: overall and body area prevalence of WMSDs among Asian nurses. WMSDs were considered according to the definition proposed by Kuorinka et al. [25]: WMSDs are symptoms such as pain and discomfort with a duration of at least one week or occurring at least once a month for the last 12 months. (5) Study design: cross-sectional study with the Nordic Musculoskeletal disorders Questionnaire (NMQ).

All body areas presented in the NMQ were assessed, i.e., neck, upper back, lower back, shoulders, elbows, wrists, hips, knees and ankles.

Studies were excluded (1) if they were not published in English; (2) if they were not a peer reviewed cross-sectional study; (3) if the study population was not Asian nurses; (4) if the sample was a mix of different healthcare professionals, with no way to differentiate between nurses and other professionals; (5) if the results did not provide the overall and by body area WMSD prevalence in percentage; and (6) if the number of assessed body areas was less than 3.

### 2.2. Quality Assessment

As the present study only included cross-sectional studies, the specific critical appraisal tool to assess the quality of cross-sectional studies (AXIS tool) proposed by Downes et al. [26] was selected to perform the quality appraisal. The Axis tool is a checklist with 20 items covering the different parts of a cross-sectional study (introduction to conclusion). Each item is evaluated according to its presence (“Yes”) or absence (“No”). The classification proposed by Hermanson and Choi [27] was chosen to assess the risk of bias: the presence of 80–100% of the AXIS tool items indicated a low risk of bias; 50–80% of items indicated a medium risk of bias; and 50% or less of the checklist items meant a high risk of bias. The two assessors (P.G. and J.J.-B.) carried out the quality appraisal separately. Then, the assessments were recorded in a spreadsheet and compared. Any discrepancies were discussed in order to reach agreement on the assessment and produce the final list. 

### 2.3. Data Extraction

The general and demographic data as well as the prevalence were extracted separately from each study. For the first set, the name of the first author, country, sample size, response rate, male/female distribution as well as the mean area, year of practice and Body Mass Index (BMI) of the nurses were recorded. The Gross Domestic Product (GDP) of the country and nurse-to-bed ratio were added for each country based on available worldwide data [28]. The ratio was obtained by dividing the number of active nurses in each country by the total number of available beds per thousand inhabitants.

Secondly, overall and by body area WMSD prevalence were reported in a table for each included study. According to NMQ, ten body areas were considered: the neck, upper back, lower back, shoulders, elbows, wrists, hips, knees and ankles. Areas with no prevalence were left blank, indicating missing data. When prevalence was formulated according to the subgroup from the main sample, it was recalculated in relation to the total sample. This conversion ensured that the results were consistent and could be compared with those of other studies.

### 2.4. Statistical Analysis

The prevalence of each body area, as well as the overall prevalence, was estimated from all available data in the included studies using the method of Neyeloff et al. [29]. Cochran’s Q test (significance level < 10%) and the I^2^ statistic were used to assess heterogeneity: 75% to 100% indicated strong heterogeneity, 50% to 90% represented substantial heterogeneity, 30% to 60% was associated with moderate heterogeneity and 0% to 40% meant weak heterogeneity [30]. To perform the meta-analysis, a fixed-effects model was used when *p* ≥ 0.1 and I^2^ ≤ 50% (no statistical heterogeneity), or a random-effects model was selected when *p* < 0.1 or I^2^ > 50% (considered to be significant heterogeneity).

To answer the two questions, “Is the prevalence of WMSD significantly influenced by the number of years of nursing experience?” and “Is there an increase in the prevalence of WMSD over the years?”, two subgroup analyses were conducted. Two groups were constructed for each one: less than and greater than 10 years’ experience as a nurse, and publication before and after 2014.

## 3. Results

### 3.1. Search Results

**Figure 2:**
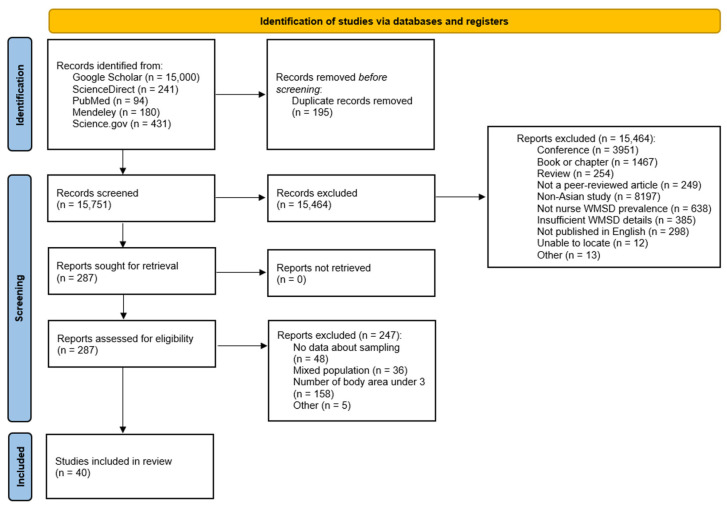
PRISMA flow diagram.

The five databases were examined and 15,946 records were identified, including 195 duplicate items. From the 15,751 unique articles, 15,464 were excluded according to the inclusion/exclusion criteria. Among the remaining 287 articles assessed from the full text, 247 were excluded because the sample or prevalence analysis by body area did not meet the selection criteria. Finally, 40 studies were included in the analysis, with a total of 19,903 nurses. The search process is depicted in Figure 2.

### 3.2. Quality Appraisal

The AXIS quality appraisal is detailed in Table 1. Twenty-six studies were assessed as having a low risk of bias (number of criteria greater than 80%) and 16 studies as having a medium risk of bias (between 50% and 80% of criteria). No study was rated with a high risk of bias.

### 3.3. Study Characteristics

**Figure 3:**
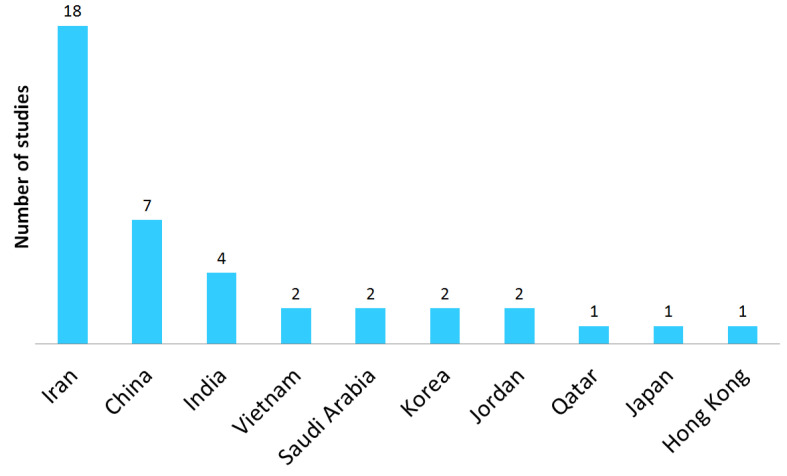
Distribution of studies by country in Asia.

The 40 studies included in the analysis were carried out in 10 different countries in Asia (Figure 3). Iran produced the highest number of studies (45.0%, 18 studies [13,18,31,32,33,34,35,36,37,38,39,40,41,42,43,44,45,46]) focusing on WMSDs in nurses, followed by China (17.5%, 7 studies [11,19,47,48,49,50,51]) and India (10.0%, 4 studies [10,52,53,54]). Two studies were conducted in Jordan, Korea [17,55], Saudi Arabia [56,57] and Vietnam [16,58]. Finally, one study was performed in Qatar [59], Japan [12] and Hong Kong [60].

**Table 1:** Quality appraisal of the included cross-sectional studies according to the AXIS tool.

	1. Were the Aims/Objectives of the Study Clear?	2. Was the Study Design Appropriate for the Stated Aim(s)?	3. Was the Sample Size Justified?	4. Was the Target/Reference Population Clearly Defined? (Is It Clear Who the Research Was About?)	5. Was the Sample Frame Taken from an Appropriate Population Base so That It Closely Represented the Target/Reference Population Under Investigation?	6. Was the Selection Process Likely to Select Subjects/Participants that Were Representative of the Target/Reference Population Under Investigation?	7. Were Measures Undertaken to Address and Categorize Non-Responders?	8. Were the Risk Factors and Outcome Variables Measured Appropriate to the Aims of the Study?	9. Were the Risk Factors and Outcome Variables Measured Correctly Using Instruments/Measurements that Had been Trialed, Piloted or Published Previously?	10. Is It Clear What was Used to Determined Statistical Significance and/or Precision Estimates? (e.g., *p* Values, CIs)	11. Were the Methods (Including Statistical Methods) Sufficiently Described to Enable Them to Be Repeated?	12. Were the Basic Data Adequately Described?	13. Does the Response Rate Raise Concerns About Non-Response Bias?	14. If Appropriate, Was Information About Non-Responders Described?	15. Were the Results Internally Consistent?	16. Were the Results for the Analyses Described in the Methods Presented?	17. Were the Authors’ Discussions and Conclusions Justified by the Results?	18. Were the Limitations of the Study Discussed?	19. Were there Any Funding Sources or Conflicts of Interest that May Have Affected the Authors’ Interpretation of the Results?	20. Was Ethical Approval or the Consent of Participants Attained?	Yes	No	Yes (%)	Risk of Bias
Abedini et al., 2013 [31]	Yes	Yes	Yes	Yes	Yes	Yes	No	Yes	Yes	Yes	Yes	Yes	No	NA	Yes	Yes	Yes	No	No	Yes	15	4	84%	Low
Akbari et al., 2017 [32]	Yes	Yes	Yes	Yes	Yes	Yes	No	Yes	Yes	Yes	Yes	Yes	No	NA	Yes	Yes	Yes	Yes	No	Yes	16	3	89%	Low
Almhdawi et al., 2020 [55]	Yes	Yes	Yes	Yes	Yes	Yes	No	Yes	Yes	Yes	Yes	Yes	No	NA	Yes	Yes	Yes	Yes	No	Yes	16	3	89%	Low
Almhdawi et al., 2021 [17]	Yes	Yes	Yes	Yes	Yes	Yes	No	Yes	Yes	Yes	Yes	Yes	No	NA	Yes	Yes	Yes	No	No	Yes	15	4	84%	Low
Anap et al., 2013 [10]	Yes	Yes	Yes	Yes	Yes	Yes	No	Yes	Yes	No	Yes	Yes	No	NA	Yes	Yes	Yes	No	No	Yes	14	5	79%	Medium
Arsalani et al., 2014 [33]	Yes	Yes	No	Yes	Yes	Yes	No	Yes	Yes	Yes	Yes	Yes	No	NA	Yes	Yes	Yes	Yes	No	Yes	15	4	84%	Low
Asghari et al., 2019 [34]	Yes	Yes	Yes	Yes	Yes	Yes	No	Yes	Yes	Yes	Yes	Yes	No	NA	Yes	Yes	Yes	No	No	Yes	15	4	84%	Low
Attar, 2014 [58]	Yes	Yes	Yes	Yes	Yes	Yes	No	Yes	Yes	Yes	Yes	Yes	No	NA	Yes	Yes	Yes	No	No	Yes	15	4	84%	Low
Attarchi et al., 2014 [35]	Yes	Yes	No	Yes	Yes	Yes	No	Yes	Yes	Yes	Yes	Yes	No	NA	Yes	Yes	Yes	No	No	Yes	14	5	79%	Medium
Barzideh et al., 2013 [36]	Yes	Yes	Yes	Yes	Yes	Yes	No	Yes	Yes	Yes	Yes	Yes	No	NA	Yes	Yes	Yes	No	No	Yes	15	4	84%	Low
Behera et al., 2023 [52]	Yes	Yes	No	Yes	Yes	Yes	No	Yes	Yes	Yes	Yes	Yes	No	NA	Yes	Yes	Yes	Yes	No	Yes	15	4	84%	Low
Chandralekha et al., 2022 [53]	Yes	Yes	Yes	Yes	Yes	Yes	No	Yes	Yes	Yes	Yes	Yes	No	NA	Yes	Yes	Yes	Yes	No	Yes	16	3	89%	Low
Choobineh et al., 2006 [13]	Yes	Yes	No	Yes	Yes	Yes	No	Yes	Yes	Yes	Yes	Yes	No	NA	Yes	Yes	Yes	No	No	Yes	14	5	79%	Medium
Choobineh et al., 2010 [37]	Yes	Yes	No	Yes	Yes	Yes	No	Yes	Yes	Yes	Yes	Yes	No	NA	Yes	Yes	Yes	No	No	Yes	14	5	79%	Medium
Dhas et al., 2023 [61]	Yes	Yes	Yes	Yes	Yes	Yes	No	Yes	Yes	Yes	Yes	Yes	No	NA	Yes	Yes	Yes	Yes	No	Yes	16	3	89%	Low
Farrokhi et al., 2016 [38]	Yes	Yes	No	Yes	Yes	Yes	No	Yes	Yes	Yes	Yes	Yes	No	NA	Yes	Yes	Yes	No	No	Yes	14	5	79%	Medium
Goswami et al., 2013 [54]	Yes	Yes	No	Yes	Yes	Yes	No	Yes	Yes	No	Yes	Yes	No	NA	Yes	Yes	Yes	No	No	Yes	13	6	74%	Medium
Heidari et al., 2018 [39]	Yes	Yes	Yes	Yes	Yes	Yes	No	Yes	Yes	Yes	Yes	Yes	No	NA	Yes	Yes	Yes	Yes	No	Yes	16	3	89%	Low
Heidari et al., 2019 [40]	Yes	Yes	Yes	Yes	Yes	Yes	No	Yes	Yes	Yes	Yes	Yes	No	NA	Yes	Yes	Yes	Yes	No	Yes	16	3	89%	Low
Hou et al., 2006 [47]	Yes	Yes	No	Yes	Yes	Yes	No	Yes	Yes	Yes	Yes	Yes	No	NA	Yes	Yes	Yes	Yes	No	Yes	15	4	84%	Low
Kee et al., 2007 [56]	Yes	Yes	No	Yes	Yes	Yes	No	Yes	Yes	Yes	Yes	Yes	No	NA	Yes	Yes	Yes	No	No	Yes	14	5	79%	Medium
Lin et al., 2020 [19]	Yes	Yes	Yes	Yes	Yes	Yes	No	Yes	Yes	Yes	Yes	Yes	No	NA	Yes	Yes	Yes	Yes	No	Yes	16	3	89%	Low
Mahmoudifar et al., 2017 [41]	Yes	Yes	No	Yes	Yes	Yes	No	Yes	Yes	Yes	Yes	Yes	No	NA	Yes	Yes	Yes	No	No	Yes	14	5	79%	Medium
Mai et al., 2022 [60]	Yes	Yes	Yes	Yes	Yes	Yes	No	Yes	Yes	Yes	Yes	Yes	No	NA	Yes	Yes	Yes	Yes	No	Yes	16	3	89%	Low
Mehrdad et al., 2010 [42]	Yes	Yes	No	Yes	Yes	Yes	No	Yes	Yes	Yes	Yes	Yes	No	NA	Yes	Yes	Yes	Yes	No	Yes	15	4	84%	Low
Mirmohammadi et al., 2014 [43]	Yes	Yes	Yes	Yes	Yes	Yes	No	Yes	Yes	Yes	Yes	Yes	No	NA	Yes	Yes	Yes	No	No	Yes	15	4	84%	Low
Nasiri-Ziba et al., 2017 [44]	Yes	Yes	No	Yes	Yes	Yes	No	Yes	Yes	Yes	Yes	Yes	No	NA	Yes	Yes	Yes	No	No	Yes	14	5	79%	Medium
Nguyen et al., 2020 [16]	Yes	Yes	No	Yes	Yes	Yes	No	Yes	Yes	Yes	Yes	Yes	No	NA	Yes	Yes	Yes	Yes	No	Yes	15	4	84%	Low
Pahlevan et al., 2014 [45]	Yes	Yes	No	Yes	Yes	Yes	No	Yes	Yes	Yes	Yes	Yes	No	NA	Yes	Yes	Yes	Yes	No	Yes	15	4	84%	Low
Shafizadeh, 2011 [18]	Yes	Yes	No	Yes	Yes	Yes	No	Yes	Yes	Yes	Yes	Yes	No	NA	Yes	Yes	Yes	No	No	Yes	14	5	79%	Medium
Smith et al., 2004 [48]	Yes	Yes	No	Yes	Yes	Yes	No	Yes	Yes	Yes	Yes	Yes	No	NA	Yes	Yes	Yes	No	No	Yes	14	5	79%	Medium
Smith et al., 2004 [49]	Yes	Yes	No	Yes	Yes	Yes	No	Yes	Yes	Yes	Yes	Yes	No	NA	Yes	Yes	Yes	Yes	No	Yes	15	4	84%	Low
Smith et al., 2005 [57]	Yes	Yes	No	Yes	Yes	Yes	No	Yes	Yes	Yes	Yes	Yes	No	NA	Yes	Yes	Yes	Yes	No	Yes	15	4	84%	Low
Smith et al., 2006 [12]	Yes	Yes	No	Yes	Yes	Yes	No	Yes	Yes	Yes	Yes	Yes	No	NA	Yes	Yes	Yes	No	No	Yes	14	5	79%	Medium
Taghinejad et al., 2016 [46]	Yes	Yes	No	Yes	Yes	Yes	No	Yes	Yes	Yes	Yes	Yes	No	NA	Yes	Yes	Yes	No	No	Yes	14	5	79%	Medium
Tariah et al., 2020 [59]	Yes	Yes	No	Yes	Yes	Yes	No	Yes	Yes	No	Yes	Yes	No	NA	Yes	Yes	Yes	No	No	Yes	13	6	74%	Medium
Yan et al., 2018 [50]	Yes	Yes	No	Yes	Yes	Yes	No	Yes	Yes	Yes	Yes	Yes	No	NA	Yes	Yes	Yes	Yes	No	Yes	15	4	84%	Low
Yang et al., 2020 [51]	Yes	Yes	No	Yes	Yes	Yes	No	Yes	Yes	Yes	Yes	Yes	No	NA	Yes	Yes	Yes	Yes	No	Yes	15	4	84%	Low
Yao et al., 2019 [11]	Yes	Yes	No	Yes	Yes	Yes	No	Yes	Yes	Yes	Yes	Yes	No	NA	Yes	Yes	Yes	Yes	No	Yes	15	4	84%	Low
Yeung et al., 2005 [62]	Yes	Yes	No	Yes	Yes	Yes	No	Yes	Yes	Yes	Yes	Yes	No	NA	Yes	Yes	Yes	Yes	No	Yes	15	4	84%	Low

NA: not applicable.

Table 2 displays the demographic data of the included studies. A wide disparity was observed for the sample sizes, ranging from 40 [54] to 3950 [47] nurses. Study populations were predominantly female. Eight studies included only females [47,48,49,52,53,59,60,61] and two studies had a higher proportion of males [32,36]. Three studies did not report this information [10,12,62]. The mean age of all studies was 31.3 ± 3.4 years. The youngest population was 21.0 ± 1.4 years old [52] and the oldest had a mean age of 37 years [40]. Twenty-eight studies had a mean sample age over 30 years. Regarding participants’ experience as nurses, the average of the studies was close to 10 years (9.4 ± 2.0 years). Eighteen studies reported experience under 10 years (the lowest value reported was 3.9 ± 4.8 years [53]), and thirteen studies had participants with an average work experience of over 10 years (with 13.8 ± 9.5 years for the most experienced nurses [49]). Nine studies did not report this information. The final information reported by the studies was the Body Mass Index (BMI), with a total mean of 22.5 ± 1.8. The 18 studies that reported BMI had a sample size considered normal (BMI between 18.5 and 25 [63]).

Table 3 summarizes the prevalence of WMSDs in Asian nurses overall and for the nine body areas. At least 30 of the 40 included studies measured the WMSD prevalence for each of the nine body areas. The three most studied areas were the neck, lower back and shoulders, with 39, 38 and 37 values available, respectively. Overall prevalence was the least studied, with 29 reported values.

**Table 2:** Country data and demographic characteristics of the samples for the 40 included studies.

Authors	Country	GDP (Billion USD)	N	Response Rate (%)	Male/Female (%)	Age (Year)	Experience (Year)	BMI	Nurse-to-Bed Ratio
Abedini et al., 2013 [31]	Iran	401.5	400	100.0%	10.2%/89.8%	30.78 ± 6.44	6.92 ± 5.75	22.78 ± 2.97	1.33
Akbari et al., 2017 [32]	Iran	401.5	220	98.3%	55.9%/44.1%	34.69 ± 6.69	10.72 ± 6.09	21.74 ± 2.95	1.33
Almhdawi et al., 2020 [55]	Jordan	50.81	597	79.6%	47.3%/52.7%	32.1 ± 5.7	9.3 ± 5.4	25.1 ± 3.8	0.82
Almhdawi et al., 2021 [17]	Jordan	50.81	597	79.6%	47.2%/52.8%	32.1 ± 5.71	9.28 ± 5.41	25.1 ± 3.77	0.82
Anap et al., 2013 [10]	India	3500	228	89.1%	-	31.4	11.5	24.06	0.6
Arsalani et al., 2014 [33]	Iran	401.5	520	92.0%	20.6%/79.4%	35.46	11.81	-	1.33
Asghari et al., 2019 [34]	Iran	401.5	147	-	19.7%/80.3%	34.6 ± 6.6	11.2 ± 6.5	24.4 ± 2.9	1.33
Attar, 2014 [58]	Saudi Arabia	1068	200	100.0%	4.5%/95.5%	34.9 ± 8.1	7.8	-	3.03
Attarchi et al., 2014 [35]	Iran	401.5	454	80.6%	24%/76%	23.2 ± 6.1	8.5 ± 3.8	24.2 ± 4	1.33
Barzideh et al., 2013 [36]	Iran	401.5	385	-	81.8%/18.2%	32.10 ± 7.3	8.4 ± 7.03	-	1.33
Behera et al., 2023 [52]	India	3500	173	70.2%	0%/100%	21 ± 1.4	-	21 ± 0.8	0.6
Chandralekha et al., 2022 [53]	India	3500	207	-	0%/100%	27.7 ± 7.3	3.9 ± 4.8	-	0.6
Choobineh et al., 2006 [13]	Iran	401.5	641	100.0%	15.3%/84.7%	32.03 ± 8.02	8.71 ± 7.77	23.01 ± 3.29	1.33
Choobineh et al., 2010 [37]	Iran	401.5	375	80.0%	33.6%/66.4%	31.5 ± 8.4	8.6 ± 7.6	22.83 ± 3.35	1.33
Dhas et al., 2023 [61]	Qatar	213	127	-	0%/100%	35.6 ± 6.12	13.0 ± 6.5	-	7.36
Farrokhi et al., 2016 [38]	Iran	401.5	250	-	19.2%/80.8%	34.56 ± 6.63	10.3 ± 6.17	19.9 ± 2.5	1.33
Goswami et al., 2013 [54]	India	3500	40	100.0%	4.5%/95.5%	27.18 ± 4.1	-	22.16 ± 252	0.6
Heidari et al., 2018 [39]	Iran	401.5	300	-	29.7%/70.3%	30.6 ± 4.4	12	-	1.33
Heidari et al., 2019 [40]	Iran	401.5	300	100.0%	29.6%/70.3%	37	12	-	1.33
Hou et al., 2006 [47]	China	17,790	3950	93.0%	0%/100%	27.63	-	-	0.8
Kee et al., 2007 [56]	Korea	1713	162	100.0%	0%/100%	29.9 ± 6.3	7.8 ± 6.0	-	0.76
Lin et al., 2020 [19]	China	17,790	1803	82.7%	0.9%/99.1%	36.63 ± 11.24	11.61 ± 9.33	-	0.8
Mahmoudifar et al., 2017 [41]	Iran	401.5	100	-	31%/69%	-	-	-	1.33
Mai et al., 2022 [60]	Vietnam	429.7	225	-	10.7%/89.3%	35.00 ± 6.39	11.9	20.51	0.44
Mehrdad et al., 2010 [42]	Iran	401.5	317	91.0%	13.3%/86.7%	33.7 ± 7.7	10.01 ± 7.6	23.26 ± 3.3	1.33
Mirmohammadi et al., 2014 [43]	Iran	401.5	120	-	33.6%/66.4%	-	-	-	1.33
Nasiri-Ziba et al., 2017 [44]	Iran	401.5	133	-	22.6%/77.4%	29.13 ± 6.8	5.84 ± 6	23.6 ± 2.7	1.33
Nguyen et al., 2020 [16]	Vietnam	429.7	1179	92.2%	18.7%/81.3%	32.5 ± 8.6	7.55	-	0.44
Pahlevan et al., 2014 [45]	Iran	401.5	286	89.0%	25.9%/74.1%	34.1 ± 7.5	7.3 ± 10.1	-	1.33
Shafizadeh, 2011 [18]	Iran	401.5	161	82.6%	13%/87%	34.9	9.32	-	1.33
Smith et al., 2004 [48]	China	17,790	282	92.0%	0%/100%	34.0 ± 9.2	13.8 ± 9.5	-	0.8
Smith et al., 2004 [49]	China	17,790	180	84.1%	0%/100%	32.7 ± 7.9	-	-	0.8
Smith et al., 2005 [57]	Korea	1713	330	97.9%	-	-	-	-	0.76
Smith et al., 2006 [12]	Japan	4213	844	74.0%	-	32.9 ± 8.8	10.0 ± 8.8	-	0.91
Taghinejad et al., 2016 [46]	Iran	401.5	135	90.0%	41.5%/58.5%	35.76 ± 8.34	-	-	1.33
Tariah et al., 2020 [59]	Saudi Arabia	1068	94	63.0%	2.1%/97.9%	34.7	-	24.12 ± 4.05	3.03
Yan et al., 2018 [50]	China	17,790	1973	90.4%	2.1%/97.9%	30.73 ± 6.58	9.01 ± 7.03	20.6 ± 3.11	0.8
Yang et al., 2020 [51]	China	17,790	679	70.7%	11.5%/88.5%	28.2 ± 4.5	6.6 ± 5.0	-	0.8
Yao et al., 2019 [11]	China	17,790	692	86.5%	5.3%/94.7%	28.3	6.9	-	0.8
Yeung et al., 2005 [62]	Hong Kong	392.1	97	100.0%	0%/100%	35.0 ± 7.0	13.5 ± 7.1	-	1.61
	**Total**	**-**	**19,903**	**88.3 ± 10.3%**	**13.3%/86.7%**	**31.3 ± 3.4**	**9.4 ± 2.0**	**22.5 ± 1.8**	**1.29 ± 1.14**

GDP: Gross Domestic Product; BMI: Body Mass Index.

**Table 3:** Overall and by body area WMSD prevalence for the 40 included studies.

Authors	Country	N	WMSD Prevalence by Body Area									WMSD Overall Prevalence
			Neck	Upper Back	Lower Back	Shoulders	Elbows	Wrists	Hips	Knees	Ankles	
Abedini et al., 2013 [31]	Iran	400	42.2%	38.8%	71.5%	42.0%	21.0%	64.5%	16.5%	35.5%	68.2%	88.2%
Akbari et al., 2017 [32]	Iran	220	50.0%	42.7%	69.1%	45.0%	31.8%	38.2%	32.7%	42.7%	29.0%	79.5%
Almhdawi et al., 2020 [55]	Jordan	597			77.4%				22.3%	37.5%	28.5%	91.0%
Almhdawi et al., 2021 [17]	Jordan	597	61.1%	47.2%		46.7%	13.9%	27.3%				91.0%
Anap et al., 2013 [10]	India	228	31.1%	10.5%	48.2%	34.6%	1.9%		1.6%	29.0%	7.6%	81.0%
Arsalani et al., 2014 [33]	Iran	520	27.0%		40.0%					35.0%		88.0%
Asghari et al., 2019 [34]	Iran	147	44.9%	32.7%	61.9%	33.3%	19.0%	31.3%	23.8%	60.5%	55.8%	92.5%
Attar, 2014 [58]	Saudi Arabia	200	20.0%		65.7%	29.0%	3.0%	10.0%	16.5%	21.0%	41.5%	85.0%
Attarchi et al., 2014 [35]	Iran	454	44.0%	47.0%	57.4%	42.0%	24.2%	37.1%	19.3%	48.4%	32.5%	
Barzideh et al., 2013 [36]	Iran	385	48.6%	54.0%	61.8%	45.5%	15.8%	48.1%	29.1%	59.7%	54.8%	89.9%
Behera et al., 2023 [52]	India	173	67.0%	62.0%	79.0%	82.0%						76.0%
Chandralekha et al., 2022 [53]	India	207	43.5%		55.1%	43.0%						81.2%
Choobineh et al., 2006 [13]	Iran	641	36.4%	46.4%	54.9%	39.8%	17.9%	39.3%	29.3%	48.4%	52.1%	84.4%
Choobineh et al., 2010 [37]	Iran	375	51.9%	54.6%	60.6%	51.7%	22.9%	47.1%	30.7%	58.1%	59.0%	85.7%
Dhas et al., 2023 [61]	Qatar	127	35.4%	29.1%	55.1%	33.9%	7.9%	17.3%	11.9%	15.0%	15.7%	90.7%
Farrokhi et al., 2016 [38]	Iran	250	58.2%		75.6%	42.0%	15.4%	40.0%		64.0%	29.4%	87.6%
Goswami et al., 2013 [54]	India	40	27.5%	6.7%	47.5%	17.5%	5.0%	15.0%	5.0%	20.0%	12.5%	
Heidari et al., 2018 [39]	Iran	300	55.0%	28.3%	83.3%	25.6%	16.3%	33.6%	71.0%	83.3%	46.0%	
Heidari et al., 2019 [40]	Iran	300	55.0%	28.3%	88.3%	25.6%	16.3%	33.6%		83.3%	46.0%	
Hou et al., 2006 [47]	China	3950	12.2%	8.0%	32.9%	17.1%	2.5%	10.5%	7.0%	6.8%	22.3%	91.6%
Kee et al., 2007 [56]	Korea	162	17.3%	12.9%	23.4%	27.2%	7.4%	21.6%	9.9%	24.7%	17.3%	56.8%
Lin et al., 2020 [19]	China	1803	62.4%	32.9%	60.4%	85.8%	53.3%	62.2%	41.7%	59.7%	47.3%	
Mahmoudifar et al., 2017 [41]	Iran	100	40.0%	37.0%	55.0%	38.0%	8.0%	11.0%	29.0%	41.0%	30.0%	
Mai et al., 2022 [60]	Vietnam	225	61.8%	35.1%	65.3%	61.8%	24.0%	34.2%	29.3%	42.2%	16.4%	87.6%
Mehrdad et al., 2010 [42]	Iran	317	46.3%	43.5%	73.2%	48.6%	16.6%	42.2%	28.8%	68.7%	39.3%	
Mirmohammadi et al., 2014 [43]	Iran	120	28.2%	17.3%						18.2%		
Nasiri-Ziba et al., 2017 [44]	Iran	133	41.4%		42.1%	34.3%	11.3%	35.3%		46.6%	29.3%	
Nguyen et al., 2020 [16]	Vietnam	1179	43.5%	31.6%	44.3%	29.0%	9.1%	16.4%	5.9%	20.0%	8.5%	74.4%
Pahlevan et al., 2014 [45]	Iran	286	65.4%	33.6%	66.1%	46.8%	13.6%	36.4%	20.3%	59.4%	36.4%	93.7%
Shafizadeh, 2011 [18]	Iran	161	62.7%	41.6%	48.4%	44.1%	14.3%	49.7%	25.5%	54.0%	37.9%	90.0%
Smith et al., 2004 [48]	China	282	45.0%	37.0%	56.0%	40.0%						70.0%
Smith et al., 2004 [49]	China	180	42.8%	38.9%	56.7%	38.9%	10.0%	27.8%	22.8%	31.1%	34.4%	70.0%
Smith et al., 2005 [57]	Korea	330	62.7%	29.7%	72.4%	74.5%	6.4%	46.7%	14.2%	35.2%	38.8%	93.6%
Smith et al., 2006 [12]	Japan	844	54.7%	33.9%	71.3%	71.9%						85.5%
Taghinejad et al., 2016 [46]	Iran	135	31.1%	23.0%	40.0%	14.1%	14.1%	11.9%	11.1%	20.0%	17.0%	71.9%
Tariah et al., 2020 [59]	Saudi Arabia	94	40.4%	48.9%	63.8%	50.0%	11.7%	34.0%	36.2%	23.4%	41.5%	
Yan et al., 2018 [50]	China	1973	57.8%		37.9%	47.4%	17.3%	23.5%	21.4%	34.1%	31.6%	79.5%
Yang et al., 2020 [51]	China	679	78.6%	39.3%	80.1%	70.4%	15.8%	38.9%	29.9%	37.4%	31.5%	97.1%
Yao et al., 2019 [11]	China	692	68.2%		39.7%	54.6%	17.3%	30.1%	23.8%	34.5%	30.6%	84.0%
Yeung et al., 2005 [62]	Hong Kong	97	19.6%	22.7%	42.3%	20.6%	7.2%	17.5%	20.6%	29.9%	19.6%	

### 3.4. Overall WMSD Prevalence—Meta-Analysis

**Figure 4:**
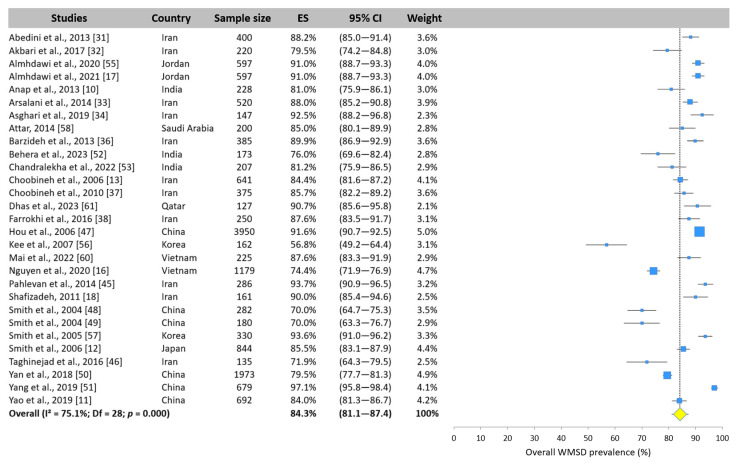
Forest plot of overall WMSD prevalence. Each blue square represents the overall prevalence. The size of the square is proportional to the sample size. The horizontal line represents the 95% confidence interval. The yellow diamond extended by the vertical dotted line represents the overall prevalence pooled across studies.

The results revealed a high heterogeneity (I^2^ > 50%). Random-effects models were applied to assess the overall prevalence of WMSDs in the meta-analysis. Twenty-nine of the forty included studies assessed the overall prevalence among nurses in Asia. This prevalence was estimated at 84.3% (95% CI: 81.1–87.4%, Figure 4). Kee et al. [61] reported a significantly lower prevalence (56.8%). Except for this study, the prevalence ranged from 70.0% [48,49] to 97.1% [51].

### 3.5. WMSD Prevalence by Body Area—Meta-Analysis

High heterogeneity (I^2^ > 50%) was observed across studies for all body areas, so random-effects models were also selected to assess prevalence. Of the nine body areas studied, lower back (58.4%), neck (45.7%) and shoulder (43.0%) were the three most affected by WMSDs. Knees ranked fourth, with a prevalence of over 40%.

#### 3.5.1. Neck WMSD Prevalence

**Figure 5:**
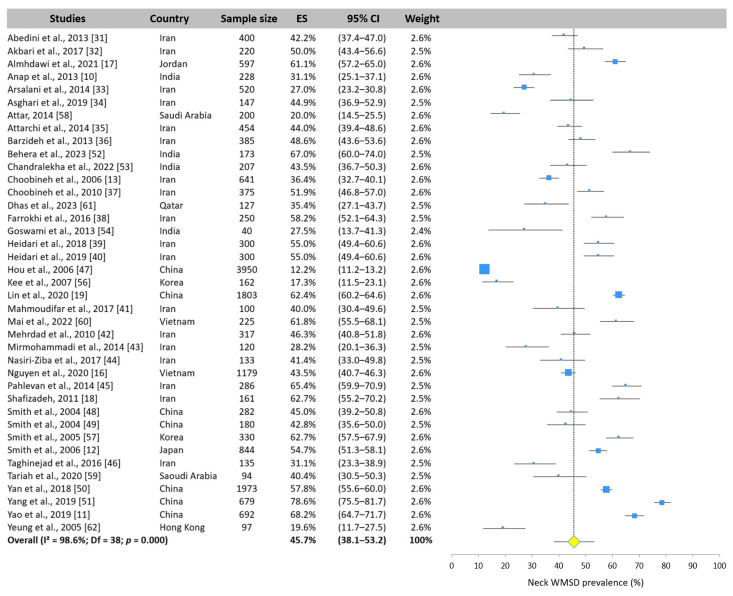
Forest plot of neck WMSD prevalence. Each blue square represents the overall prevalence. The size of the square is proportional to the sample size. The horizontal line represents the 95% confidence interval. The yellow diamond extended by the vertical dotted line represents the neck WMSD prevalence pooled across studies.

The neck was the most studied area. Only one of the 40 selected studies did not report the prevalence. The meta-analysis revealed that the neck was the second body area most affected by WMSDs, with a prevalence of 45.7% (95% CI: 38.1–53.2%). However, there was considerable disparity between studies, with the prevalence ranging from 12.2% [47] to 78.6% [51] (Figure 5).

#### 3.5.2. Upper Back WMSD Prevalence

**Figure 6:**
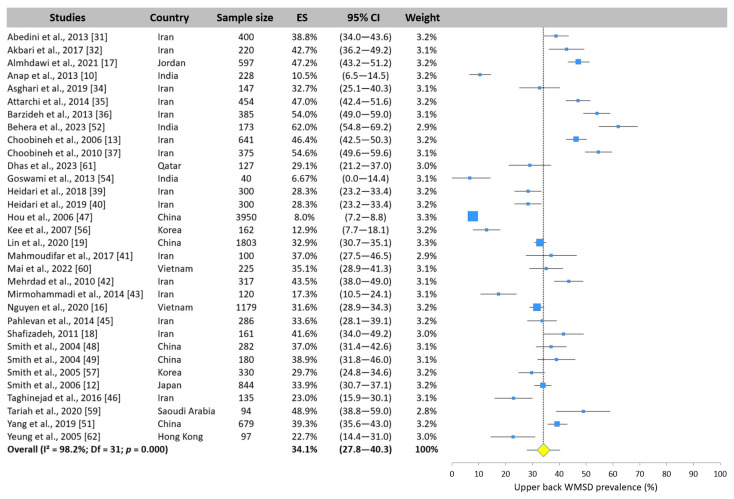
Forest plot of upper back WMSD prevalence. Each blue square represents the overall prevalence. The size of the square is proportional to the sample size. The horizontal line represents the 95% confidence interval. The yellow diamond extended by the vertical dotted line represents the upper back prevalence pooled across studies.

Thirty-two studies assessed the upper back WMSD prevalence. The results reported by the different cross-sectional studies differed widely, with values ranging from 6.7% [54] to 62.0% [52] (Figure 6). The meta-analysis showed that one nurse in three (34.1%, 95% CI: 27.8–40.3%) experienced an upper back WMSD in Asia.

#### 3.5.3. Lower Back WMSD Prevalence

**Figure 7:**
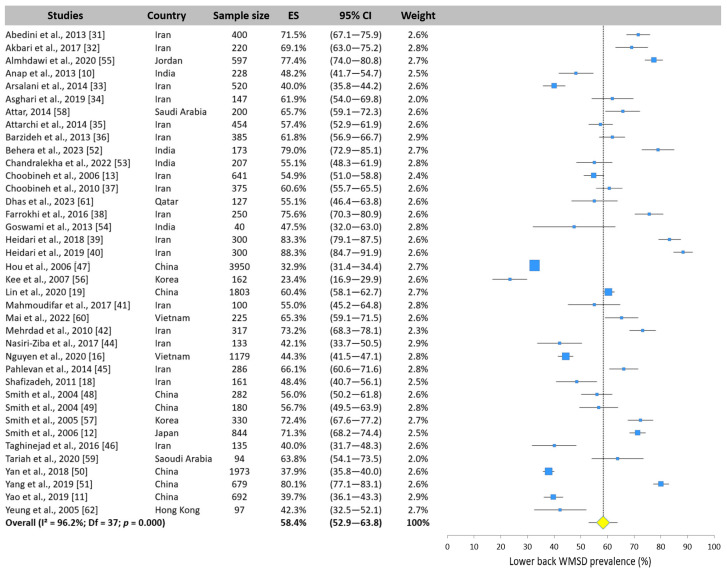
Forest plot of lower back WMSD prevalence. Each blue square represents the overall prevalence. The size of the square is proportional to the sample size. The horizontal line represents the 95% confidence interval. The yellow diamond extended by the vertical dotted line represents the lower back prevalence pooled across studies.

The lower back has been extensively studied in the literature. Thirty-eight of the forty included studies assessed the prevalence of WMSDs using the NMQ. Figure 7 displays the values reported by the different studies, which ranged from 23.4% [61] to 88.3% [40]. The lower back was the area with the highest prevalence among Asian nurses, with a mean value of 58.4% (95% CI: 52.9–63.8%).

#### 3.5.4. Shoulder WMSD Prevalence

**Figure 8:**
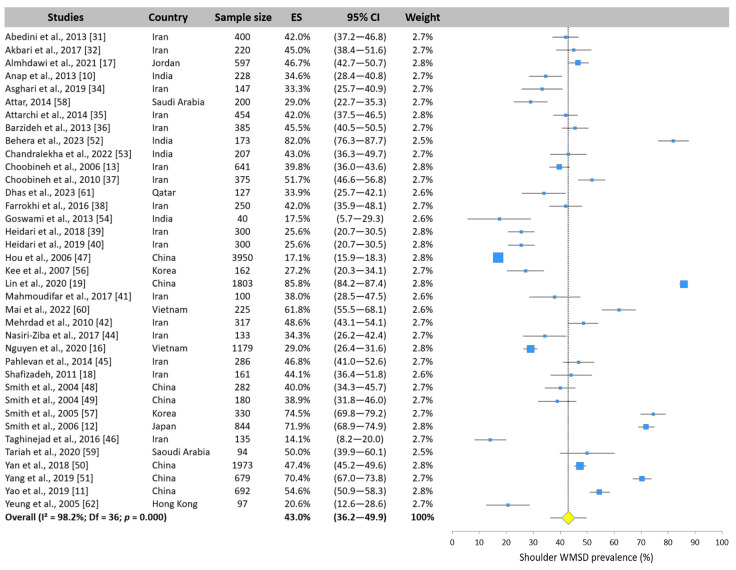
Forest plot of shoulder WMSD prevalence. Each blue square represents the overall prevalence. The size of the square is proportional to the sample size. The horizontal line represents the 95% confidence interval. The yellow diamond extended by the vertical dotted line represents the shoulder prevalence pooled across studies.

The shoulder was the third most prevalent body area and the upper limb body area most exposed to WMSD in Asian nurses. The 37 values reported by the surveys ranged from 14.1% [46] to 85.8% [19] (Figure 8). The meta-analysis pooled a mean prevalence of 43.0% (95% CI: 36.2–49.9%).

#### 3.5.5. Elbow WMSD Prevalence

**Figure 9:**
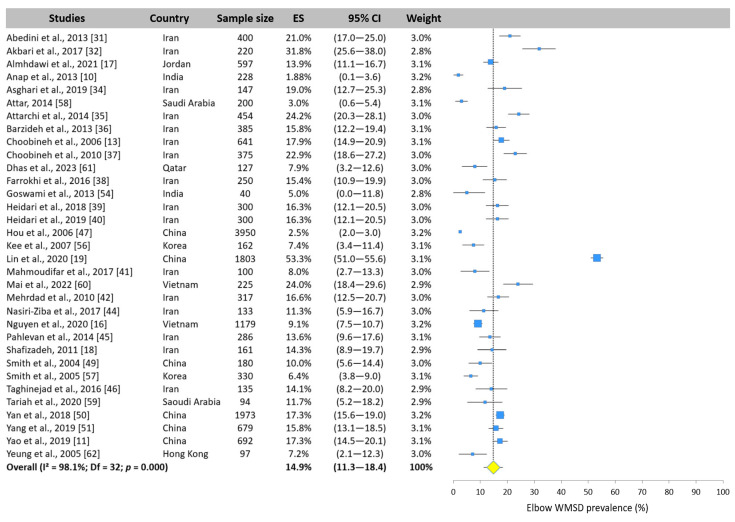
Forest plot of elbow WMSD prevalence. Each blue square represents the overall prevalence. The size of the square is proportional to the sample size. The horizontal line represents the 95% confidence interval. The yellow diamond extended by the vertical dotted line represents the elbow prevalence pooled across studies.

The meta-analysis revealed that the elbow was the body area least exposed to WMSDs. The 33 values reported ranged from 1.9% [10] to 53.3% [19] with a pooled mean value of 14.9% (95% CI: 11.3–18.4%, Figure 9).

#### 3.5.6. Wrist WMSD Prevalence

**Figure 10:**
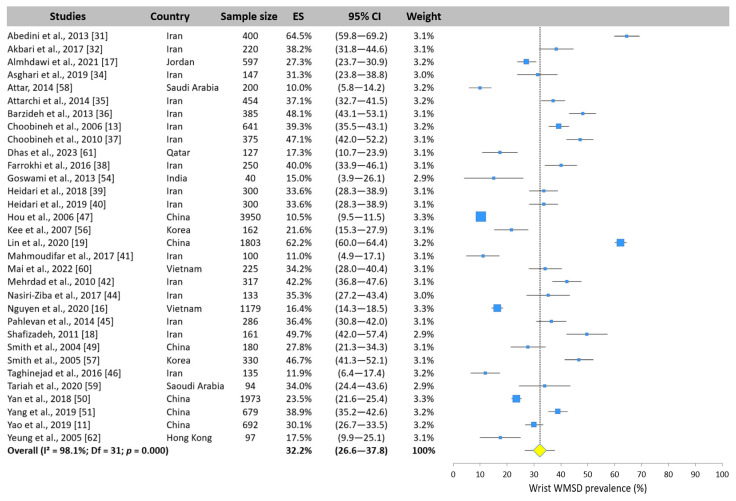
Forest plot of wrist WMSD prevalence. Each blue square represents the overall prevalence. The size of the square is proportional to the sample size. The horizontal line represents the 95% confidence interval. The yellow diamond extended by the vertical dotted line represents the wrist prevalence pooled across studies.

The WMSD prevalence in the wrist was assessed in 32 studies. The overall prevalence was 32.2% (95% CI: 26.6–37.8%). Reported values ranged from 10% [56] to 64.5% [31] (Figure 10).

#### 3.5.7. Hip WMSD Prevalence

**Figure 11:**
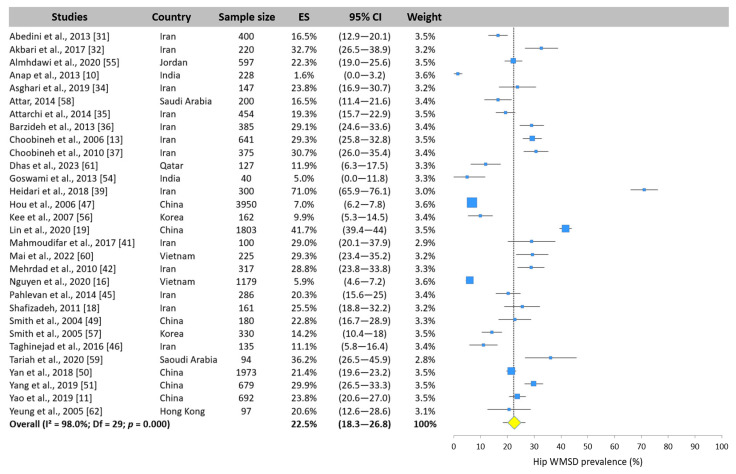
Forest plot of hip WMSD prevalence. Each blue square represents the overall prevalence. The size of the square is proportional to the sample size. The horizontal line represents the 95% confidence interval. The yellow diamond extended by the vertical dotted line represents the hip prevalence pooled across studies.

The analysis showed that the hip was the second most and least affected lower limb area for WMSDs after the elbow. The overall prevalence was 22.5% (95% CI: 18.3–26.8%) based on the 30 values from the 40 included studies. Five studies reported a prevalence less than 10%, with the lowest value recorded by Anap et al. [10] at 1.6%. Heidari et al. [39] reported a very high value compared with the other studies, with a prevalence of 71.0% (Figure 11).

#### 3.5.8. Knee WMSD Prevalence

**Figure 12:**
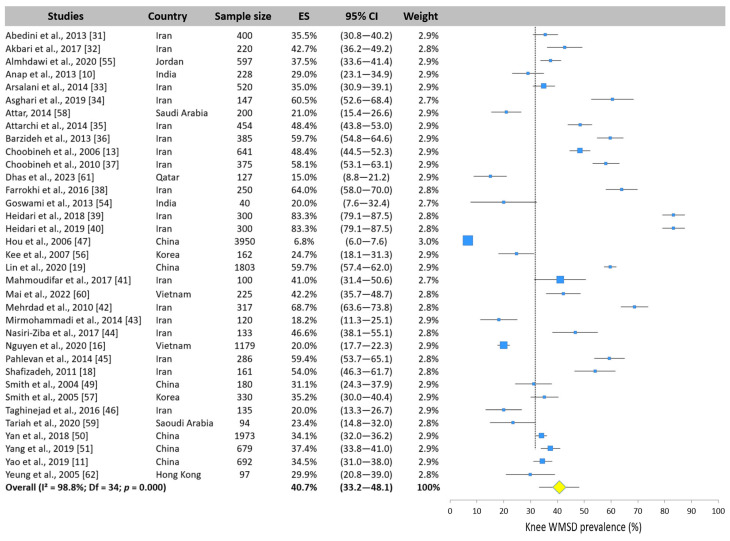
Forest plot of knee WMSD prevalence. Each blue square represents the overall prevalence. The size of the square is proportional to the sample size. The horizontal line represents the 95% confidence interval. The yellow diamond extended by the vertical dotted line represents the knee prevalence pooled across studies.

Thirty-five studies investigated the prevalence of knee WMSDs. The results highlighted that the knee was the most exposed area of the lower limb and was the fourth most exposed area to WMSDs overall. The pooled value across the studies was 40.7% (95% CI: 33.2–48.1%). The extremums were extremely distant. Indeed, the study by Hou et al. [47] reported a very low prevalence of 6.8%, while Heidari et al. [39,40] reported a value twelve times higher (83.3%, Figure 12).

#### 3.5.9. Ankle WMSD Prevalence

**Figure 13:**
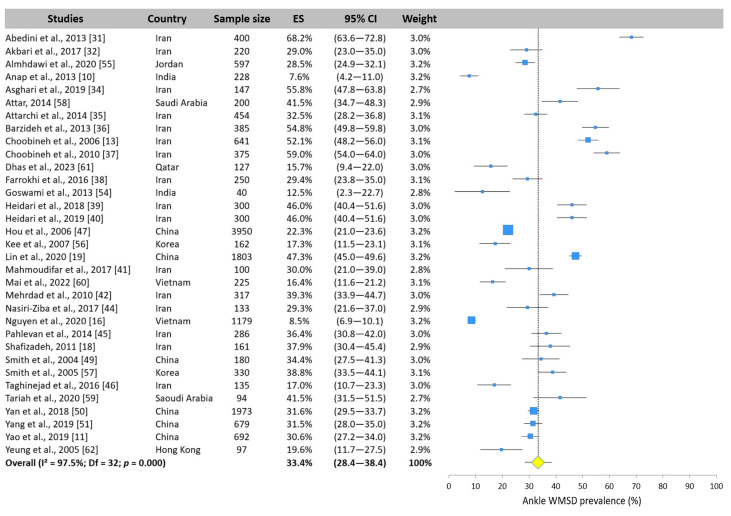
Forest plot of ankle WMSD prevalence. Each blue square represents the overall prevalence. The size of the square is proportional to the sample size. The horizontal line represents the 95% confidence interval. The yellow diamond extended by the vertical dotted line represents the ankle prevalence pooled across studies.

The WMSD prevalence of the ankle was reported by 33 studies and is displayed in Figure 13. Values ranged from 7.6% [10] to 68.2% [31]. The pooled value across the meta-analysis was 33.4% (95% CI: 28.4–38.4%).

### 3.6. WMSD Subgroup Meta-Analysis

Table 4 shows the prevalence by body area and year of experience. The overall prevalence is very high, but is reduced by 1% for the most experienced group. Across all nine body areas, the prevalence of five of them (lower back, elbow, wrist, hip, knee) increased with experience. The lower back and knee were the two areas with the highest increase (7−10%). Conversely, the upper back and ankle showed the largest decrease in prevalence (5 to 9%).

The analysis of groups by year of publication demonstrated that seven of the nine body areas presented an increasing prevalence over the years (between 4.5% and 13%). In contrast, the extremities of the upper and lower limbs (wrist and ankle) experienced a lower prevalence in the most recent studies, at around 5% (Table 5).

## 4. Discussion

The aim of this study was to propose a systematic review and meta-analysis to investigate the prevalence of WMSDs among nurses in Asia. The objective was to investigate the overall prevalence of WMSDs and the prevalence by body area. The effect of experience and year of publication were added to improve WMSD analysis. Forty studies were identified and included in the present study.

Overall prevalence was the first indicator of the presence of WMSDs in nurses. The results were highly heterogeneous, with values ranging from 70.0% to 97.1%. The meta-analysis provided an overall prevalence for Asia of 84.3% based on the 28 values reported. This result is slightly lower than the prevalence measured in Europe (87.8% [23]), but higher than the worldwide trend (71.85% [64] and 77.2% [7]). This indicates that nurses as healthcare professionals widely exposed to WMSDs. There are several possible reasons for such a high prevalence. Firstly, nurses are responsible for a large number of patients, for whom they must provide a high number of care treatments throughout their shift. This physical and cognitive workload imposes heavy demands on the body for many consecutive hours, and contributes to the development of WMSDs [65]. In addition, shift rotations are inherent to uninterrupted care in healthcare sector facilities. These rotating or irregular shifts alter the quality of rest and further increase the risk of WMSDs [66].

The second indicator was the analysis of prevalence by body area. The meta-analysis revealed that the area most exposed to WMSDs among Asian nurses was the lower back (58.4%). This result is in line with the international literature synthesized through national (Iran: 60.0% [15]), continental (Europe: 61.4% [23]) and worldwide (62.0% [2]) meta-analyses. The neck (45.7%) and shoulder (43.0%) were the second and third most affected body areas in Asia. This ranking is comparable to the worldwide analysis proposed by Sun et al. [7], but differs from other rankings proposed in Europe [23] or in other worldwide analyses [2,67]. Several factors may explain these differences in ranking. First of all, not all nurses have the same physical constraints, depending on their department. Some departments require specific postures which may expose some parts of the body or specific body areas. For example, Tavakkol et al. [67] and Clari et al. [2] studied groups of specialized nurses (operating room nurses and perioperative nurses, respectively). In both studies, the knee or ankle was the second most exposed area to WMSDs. This is linked to the static standing postures maintained for long periods, which are not found in other departments. In departments involving more displacements and repeated tasks, the neck, upper back and shoulders are more exposed. Whatever the classification, the areas most affected have a prevalence of over 40%, indicating high exposure to WMSDs. Several studies have investigated the various risk factors responsible for this high prevalence among nurses. Working in awkward positions and in the same position for long periods (standing, bending, sitting, kneeling, etc.), performing the same task repeatedly or performing a task very quickly for short periods (lifting, gripping, pulling, pushing, etc.) [55,68] are the risk factors most often reported to cause WMSDs. They are observed during the numerous daily manipulations of patients (transfer and repositioning [68,69]) or equipment [70], and during multiple acts of care (measurement of vital signs, administration of medication, etc. [71]).

A regional subgroup analysis was carried out on the annual WMSD prevalence for all body areas. The results showed that for half of the body areas (lower back, elbow, wrist, hip, knee), the prevalence decreased with years of experience, while it increased for the other areas. Despite professional experience and advanced age, the lower back was one of the areas where prevalence increased. This was explained by nurses’ daily activities. Many of these care activities (repositioning, transferring and helping to move the patient, measuring vital signs, administering medication) involve the repeated and prolonged flexion of the trunk with rotation and inclination [72,73]. As a result, nurses adopt awkward postures, which are a major risk factor in the occurrence of WMSDs. Not surprisingly, the literature points out that this is the area most affected. For the shoulder and neck, which are highly exposed to WMSDs, the prevalence tends to decrease with experience. It is possible that nurses protect these areas either after exposure to repeated pain or by adapting their posture and practice to preserve these zones. However, this exposes the lower back to more WMSDs. Secondly, the temporal subgroup analysis was performed. The results showed that over the past 20 years, the WMSD prevalence increased for all body areas except for the extremities of the upper and lower limbs. This result is in line with the findings reported worldwide [7]. This means that, despite the implementation of programs to prevent the occurrence of WMSDs, such as increasing the nurse-to-bed ratio [9], the prevalence continues to rise. Thus, the working conditions of nurses in Asia still need to be improved to control this increase. Several points can be raised and considered in the development of future health policies. On the one hand, the high prevalence of WMSDs is linked to numerous awkward postures and repeated patient handling during the course of the day. One solution is to ensure that the settings of the various equipment—beds, perfusion supports, transport carts, etc.—allow ergonomic handling that minimizes the risk of WMSDs. On the other hand, in the organization of work, the reduction in work constraints, such as distance traveled and stress levels, could be envisaged with an increase in the number of nurses per bed. The introduction of regular physical activity in the workplace is also a solution for reducing the occurrence of WMSDs [11,74]. However, it is difficult to generalize these results from these preliminary observations, given the small number of studies that have addressed this issue. Further work is needed to find more specific explanations and propose more appropriate solutions for nurses.

Some limitations could be addressed. The first concerns data availability. Despite the selection of articles that collected data using the NMQ, some prevalence data were not present in all works. A second limitation is the choice to include all nurse profiles in the study. Indeed, factors such as department or nurse profile can affect the results obtained, as shown by the great variability observed between studies. The final limitation concerns the language used. Indeed, several studies carried out in Asia were published only in Chinese and therefore could not be included in the analysis.

## 5. Conclusions

Asian nurses are highly exposed to WMSDs (84.3%). The lower back, neck and shoulder were identified by the meta-analysis as the areas most affected by WMSDs, with the knees being most prevalent for the lower limbs. Subgroup analyses showed that the presence of WMSDs is steadily increasing, and that years of practice reduce the exposure of some areas (notably the neck and shoulder), while others, such as the lower back, are more exposed. Sub-group analyses by specialty or demographic characteristics would provide a better understanding and an explanation of the high incidence of WMSDs. Further efforts are needed to prevent WMSDs among Asian nurses in order to improve their quality of life at work.

## Figures and Tables

**Table 4:** Meta-analysis results of WMSD prevalence by body area in relation to year of experience.

Body Area	Experience < 10 Years					Experience > 10 Years				
	N	Sample Size	I^2^	ES	95% CI	N	Sample Size	I^2^	ES	95% CI
Neck	17	9368	96.0%	49.2%	(41.9–56.4)	13	4816	94.7%	45.5%	(36.7–54.2)
Upper back	12	6163	93.4%	39.9%	(33.8–46.0)	11	4046	91.5%	31.0%	(24.7–37.3)
Lower back	17	9368	96.1%	56.3%	(48.6–63.9)	13	4816	91.0%	62.8%	(55.0–70.7)
Shoulder	17	9368	94.9%	45.1%	(38.9–51.3)	12	4296	97.8%	41.4%	(27.4–55.4)
Elbow	15	8317	92.5%	14.9%	(11.9–17.9)	11	4014	98.7%	19.0%	(8.0–30.0)
Wrist	15	8317	96.5%	34.6%	(28.3–40.9)	10	3786	95.9%	35.1%	(24.6–45.7)
Hip	14	8184	96.8%	21.4%	(16.1–26.6)	9	3464	98.9%	28.9%	(13.7–44.2)
Knee	15	8317	95.6%	40.9%	(34.5–47.2)	12	4534	96.6%	50.9%	(39.3–62.5)
Ankle	15	8317	98.3%	37.1%	(28.3–46.0)	11	4014	97.3%	31.8%	(21.0–42.6)
Overall	16	9378	77.0%	84.8%	(80.7–89.0)	8	1999	42.1%	83.8%	(78.4–89.2)

N: the number of studies; ES: effect sizes computed using a random-effects model due to significant heterogeneity; 95% CI: 95% confidence interval.

**Table 5:** Meta-analysis results of WMSD prevalence by body area in relation to year of publication.

Body Area	2004–2014					2015–2024				
	N	Sample Size	I^2^	ES	95% CI	N	Sample Size	I^2^	ES	95% CI
Neck	20	9972	98.2%	39.2%	(30.3–48.1)	19	9334	90.8%	52.8%	(47.5–58.1)
Upper back	18	9252	98.4%	32.0%	(23.1–40.9)	14	6079	82.7%	36.1%	(32.1–40.1)
Lower back	19	9852	96.4%	55.3%	(47.2–63.4)	19	9334	95.8%	61.5%	(53.8–69.3)
Shoulder	18	9332	97.9%	40.7%	(31.4–50.0)	19	9334	97.7%	45.3%	(36.1–54.5)
Elbow	16	8206	96.3%	11.7%	(8.2–15.3)	17	8954	97.2%	17.8%	(12.4–23.2)
Wrist	15	7978	98.2%	34.2%	(24.3–44.1)	17	8954	97.2%	30.5%	(23.6–37.4)
Hip	16	8206	96.7%	18.4%	(13.7–23.1)	14	8271	98.3%	27.5%	(19.7–35.3)
Knee	18	8846	98.7%	37.9%	(26.7–49.1)	17	8954	97.3%	43.4%	(35.3–51.5)
Ankle	16	8206	97.1%	35.8%	(27.9–43.8)	17	8954	97.8%	31.1%	(23.7–38.6)
Overall	15	8944	76.5%	84.0%	(79.4–88.5)	14	7201	70.7%	84.5%	(80.1–88.8)

N: the number of studies; ES: effect sizes computed using a random-effects model due to significant heterogeneity; 95% CI: 95% confidence interval.

## Data Availability

Data available on request.
